# Filling the sustainability gap: what beef industry stakeholders can learn from ranchers on new practice adoption, grazing management plans, and sustainability

**DOI:** 10.1093/tas/txaf045

**Published:** 2025-04-16

**Authors:** S C Klopatek, A M Cantwell, L Roche, J W Oltjen

**Affiliations:** Nelson Institute for Environmental Studies, University of Wisconsin-Madison, Madison, WI 95616, USA; Sarah Klopatek serves as JBS' Global Chief Livestock Scientist and is a UW Nelson Institute for Environmental Studies Honorary Fellow; Department of Sociology, Colorado State University, Fort Collins, CO 80523, USA; Department of Plant Sciences, University of California, Davis, CA 95616, USA; Department of Animal Science, University of California, Davis, CA 95616, USA

**Keywords:** sustainability, cattle, survey, grazing management, Scope 3

## Abstract

Livestock’s environmental footprint has become a pivotal concern for consumers and food corporations alike. To stay competitive in the sustainable foods movement and reduce Scope 3 emissions, the beef industry has sought to improve sustainability within their own value chains. However, before system sustainability can be achieved, sustainability practices must be adopted and implemented by the foundation of the beef system, cow-calf operations. Therefore, to gain insight into ranchers’ motivations for adopting new sustainable practices we administered an online multi-state survey to cattle ranchers in collaboration with state cattlemen’s associations. The survey objectives were to: 1) identify where ranchers obtain educational information, 2) identify ranching priorities and reasons for new practice adoption, 3) determine factors influencing grazing management plan adoption, and 4) assess what sustainability means to the ranching community. Of the survey participants who fully completed the survey (n=706), when asked what made them trust in a ranching educational program, 64% of ranchers selected science, 55% selected programs partnered with cattlemen associations, and 32% trusted the program if other ranchers they knew were enrolled in the program. Increased profitability (79%), animal health (78%), and expected benefits would outweigh costs (51%) were key drivers for ranchers adopting a new practice. A top priority/principal concern for ranchers was improving ranch public image (n= 551), likely influenced by public rhetoric and concerns around beef’s environmental impact. In terms of grazing management plans, 77% of producers stated they had a grazing management plan and 38% of ranchers indicated their plan was written. The likelihood of having a grazing management plan increased with rancher age, presence of a succession plan, and participation in a land assistantship program (*P* < 0.01). In total, 114 survey participants answered the open-ended question, “What does sustainable ranching mean to you.” Based on responses, ranchers' definition of sustainability was a multifaceted concept balancing environmental health, profitability, family, and animal welfare, with many viewing these elements as interconnected and critical for the future of their operations. In conclusion, the information accrued in this survey provided guidance to beef industry stakeholders on how to effectively engage ranchers and encourage the broader adoption of sustainable management practices and programs.

## Teaser

If beef industry stakeholders want to increase participation in their sustainability programs they need to hear directly from ranchers. So we asked ranchers across the country, “What does sustainability mean to you?”

## Graphical Abstract

**Graphical Abstract:**
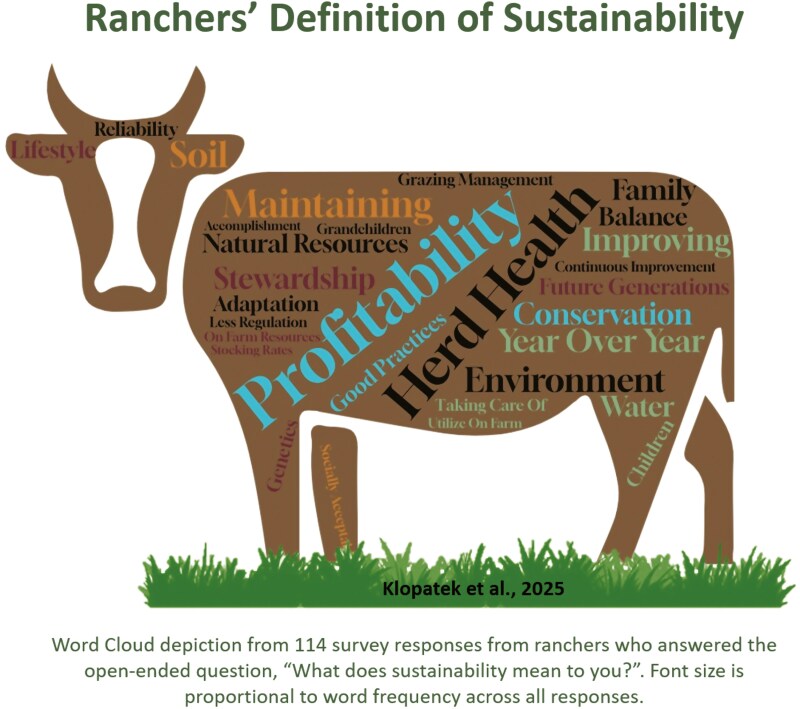

## INTRODUCTION

The discussion of Scope 3 greenhouse gas (GHG) emissions—covering companies’ downstream and upstream emissions—has steamrolled from academic institutions into C-suite boardrooms^1^. Globally, from 2010 to 2021, the number of companies reporting Scope 3 GHG emissions—either publicly or privately—increased by over 740% (from 936 companies to 7,000; Hadziosmanovic et al., 2022). Additionally, more than 90% of Fortune 500 companies have committed to reducing Scope 3 emissions (EY, 2022). In the Food and Agriculture sector, beef has one of the highest carbon intensities, prompting a surge of funding for carbon reduction projects, principally within the cow-calf grazing sector. Despite growing interest and funding in the beef sector, considerable gaps persist between industry carbon reduction goals and rancher willingness to adopt specific sustainability practices. Understanding how ranchers^2^ learn about sustainability programs and where they obtain their information could help bridge these gaps (Ahlering et al., 2023), ultimately strengthening the effectiveness and adoption of a sustainability program. Without this understanding, it will be difficult to attract ranchers to both educational programs and corporate sustainability initiatives.

While limited research has directly explored why ranchers join sustainability programs, numerous studies have investigated rancher motivations for participating in conservation programs (Lubell et al., 2013; Brain et al., 2014; Roche et al., 2015). Previous research has highlighted the importance of financial remuneration for practice adoption (Victurine and Curtin, 2010), the necessity of rancher trust in programs (Lubell et al., 2013; Brain et al., 2014), and the need for program flexibility to enhance adoption of environmentally focused initiatives (Roche et al., 2015). Furthermore, studies have found that ranchers are more willing to participate in conservation programs if they believe the practices will improve ranch resilience and support ranch succession (Brain et al., 2014; Lien et al., 2017). The continued rancher interest and involvement in conservation programs suggests that ranchers may have similar interests in joining value chain sustainability initiatives (Ahlering et al., 2023). While insights can be gained from studying rancher motivations for joining conservation programs, it is important to recognize the fundamental differences between these programs. Corporate sustainability programs are in the private sector and are generally outcome based that pay producers based on key performance indicators. These include finical and operating metrics. In contrast, conservation programs are predominantly in the public sector and are more focused on land-based activity and cost sharing arrangements. Therefore, there is a need for investigation into the distinct motivations driving ranchers to engage in sustainability-oriented programs.

One best management practice that has become integral to both conservation and sustainability programs has been the implementation of grazing management plans (GMP; USDA-NRCS 2022; USRSB, 2023). Grazing management plans are essential for managing natural resources, optimizing animal productivity, and ensuring ranch financial success (Follett and Reed, 2010). While maintaining a GMP does not guarantee improvements in ecological or economic outcomes, maintaining a plan can be a foundational step for achieving these positive outcomes. In 2018, the United States Roundtable for Sustainable Beef (USRSB), recognizing the importance of GMP, set a goal to have 385 million acres (156 million hectares) covered by a written GMP by 2050 (USRSB, 2023). In terms of corporate interest, effective grazing management has been identified as a potential GHG mitigation strategy (Follett and Reed, 2010), which can possibly aid companies in both reducing their Scope 3 impacts and improving value chain resilience. Although surveys have investigated GMP at a regional level (Bruno et al., 2022; Banwarth et al., 2023; Jablonski et al., 2024), there has been limited data available at the national level investigating if ranchers have a GMP, if the GMP is written, and what influenced ranchers to adopt a GMP. Knowing more about GMP motivations and plan development can aid the beef value chain in increasing GMP adoption. Furthermore, the development and monitoring of GMP effectiveness could be instrumental for building successful sustainability programs and aid stakeholders in achieving their own beef sustainability goals.

To bridge the gaps between the ranching community and corporate sustainability programs we expanded our national rancher BQA survey (Klopatek et al., 2022) to include sustainability. The survey objectives were to: 1) identify where ranchers obtain educational information, 2) identify reasons for new practice adoption and ranching priorities, 3) determine factors influencing grazing management plan adoption, and 4) assess what sustainability means to the ranching community.

## MATERIALS AND METHODS

### Survey Design and Recruitment Procedures

**Figure 1.**
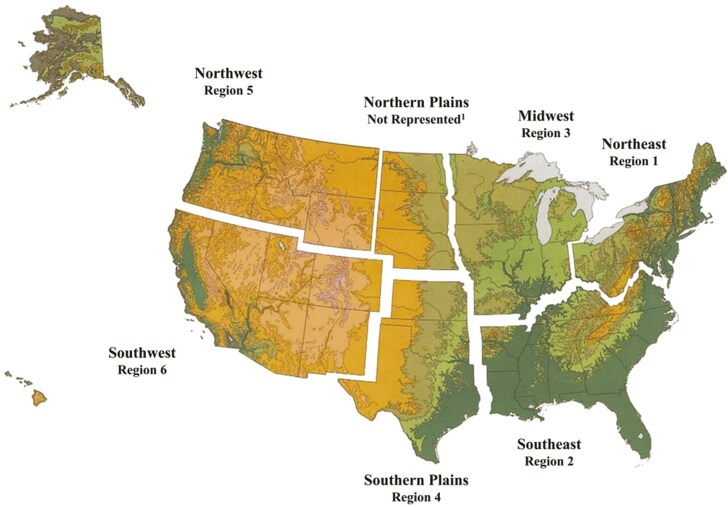
The “Filling the Sustainability Gap” survey was administered to ranchers across six of the seven National Cattlemen’s Beef Association Rancher regions in the United States. The survey was administered from June 1^st^ to December 31^st^, 2019. ^1^Image provided by the National Beef Cattlemen’s Association 2 Ranchers represented in Northwest Region (Region 1), Northern Plains, Midwest Region, Northeast Region, Southwest Region (Region 6: 288), Southeast Region, and southern Plains.

An online survey for ranchers was developed and administered using the Qualtrics platform (Provo, UT). In total, the survey consisted of 45 questions divided into four sections: 1) rancher demographics, 2) ranching educational resources, 3) ranch management and Beef Quality Assurance practices, and 4) willingness to join a sustainability program. Results related to Beef Quality Assurance practices were published in Klopatek et al. (2022). Survey questions were derived from literature and discussions with collaborating partners. After question development, the initial survey was pilot tested with a group of northern California ranchers. Once pilot testing was completed and necessary adjustments made, the final survey was administered online to ranchers across six of the seven National Cattlemen’s Beef Association (NCBA) regions, specifically the Northwest, Southwest, Southeast, Midwest, Northern Plains, and Southern Plains (Klopatek et al., 2022; Figure 1). This strategy ensured the survey would capture ranchers with diverse perspectives and management approaches. To recruit ranchers from these regions, ranchers were contacted through state cattlemen association listservs. The state cattlemen associations are nonprofit trade organizations serving cattle ranchers, beef producers, and private owners of cattle-grazed properties. Specifically, cattlemen’s associations serve as an in person and online resource where ranchers receive information and provide feedback on ranching practices and policies. Ranchers on the respective listservs were emailed once a month over a 6-mo period with an invitation to complete the survey. The survey was available from June 1^st^ to December 31^st^, 2019. Participants were enrolled in the study if they: 1) were a cattle rancher on a state cattlemen listserv, and 2) owned cattle at the time of survey distribution. Although ranchers who did not have access to the internet were excluded, recent studies have determined that internet use is widespread among today’s ranchers (75% to 82%; Kachergis et al, 2013; Ghajar et al., 2019). The survey was administered to 1,000 ranchers and 706 answered all questions included in this analysis for the exception of the fill in the blank, “What does sustainability mean to you” where 114 participants answered the questioned.

### Operator and Operation Characteristics

To provide insights into the key factors shaping rancher adoption of management practices, descriptive statistics were used to characterize key components adapted from the rangeland decision-making framework of Lubell et al. (2013). Summary information about survey participants including region of operation, sex, age, first or multi-generational rancher, years of ranching since age 18 are included in Table 1. Operator characteristics included for this study were percent of income from ranching, existence of a succession plan, number of head of cattle, type of land managed (leased, owned, private, or public land) and total acres grazed (Table 1). In addition, ranchers were asked about common rangeland and ranch management practices, type of operation, types of certifications, program involvement, vegetation management, and landscape enhancements. Quantitative questions were multiple choice and qualitative questions were fill in the blank. Please see supplemental material for the full list of survey questions.

**Table 1.** Summary Statistics used in regression analysis (n = 706).

Categorical Variables	Frequency	Percent
Age		
18-29	68	9.62
30-39	127	17.9
40-49	103	14.5
50-59	155	22.2
60-69	162	22.8
Over 70	91	13.0
Years ranching (Since 18 yr old)		
0-5	83	11.8
6-10	89	12.6
11-20	144	20.4
21-30	129	18.3
More than 30 yr	261	36.9
Gender		
Male	523	74.1
Female	183	25.9
NCBA^1^ region		
1-Northeast	303	42.9
2-Southeast	44	6.18
3-Midwest	22	3.14
4-Southern plains	46	6.53
5-Northwest	79	11.22
6-Southwest	212	30.03
Succession plan		
Yes (Includes “in progress”)	236	33.4
No	470	66.6
Generation of ranching		
First	230	32.7
Multi-generational	476	67.4
Percentage of income from ranching		
1-25%	373	52.8
26-50%	173	24.5
51-75%	73	10.3
76-100%	87	12.4
Programs: Humanely Raised, Verified Source and Age, NHTC, GAP		
Participating	204	28.9
Not Participating	502	71.1
Programs: Grass Fed, All Natural, and/or Certified Organic		
Participating	123	17.3
Not Participating	583	82.7
Participate in a government landowners assistance program		
Yes	421	59.7
No	285	40.3

^1^National Cattlemen’s Beef Association.

^2^Examples of land management programs included USDA or Natural Resource Conservation Service (NRCS), such as the Environmental Quality Incentives Program (EQIP), Conservation Stewardship Program (CSP)].

### Ranch Management Practices

To gain insight into rancher’s behavior, we asked about trusted sources of information regarding ranching. Survey participants answered questions regarding which organizations or entities they trusted for ranching information, and the types of information they considered reliable. Each participant rated their trust in each information source on a 5-point scale, ranging from “extremely trustworthy” to “not trustworthy.” They were also asked how frequently they acquired information from each source, using a 5-point scale ranging from “always utilize” to “never utilize.” To identify opportunities and barriers to adoption, we asked survey participants to select up to 3 reasons for adopting or not adopting a practice or program. The list of options for participants to choose as a reason for not adopting a ranching practice or program included: It would require more time investment, Does not fit your production system, Requires certification, Requires re-certification, Requires audits, Expected cost outweighs expected benefits, Benefits are short term (5 yr or less), Does not align with your values, and Other. Finally, participants were asked to choose their top two ranching priorities from an option of eight priorities, with “other” being an optional category. The list of options for participants to choose from for the question what is your top ranching priority included: Improving/maintaining cow nutrition (ex. body condition score); Improving/maintaining fertility rates, Improving/maintaining forage productivity, Improving/maintaining herd health, Improving/maintaining genetics of the herd, Improving/maintaining ranch publicity/ public image, and Reducing additional forage supplementation.

### Statistical Analysis

All analyses were conducted using Stata version 16.1 (StataCorp 2019). The frequency tables for variables in all models were summarized in Table 2. To determine if demographic factors influenced a rancher’s primary ranching concern a logistic regression was run against each survey response category (response variables were either yes or no for each response category). The regression model used in the present study was identical to the one used in Klopatek et al., 2022. Logistic regression was used again to identify what demographic factors best predicted whether ranchers maintained a grazing management plan, and if the GMP was written. Response categories for these questions was either “yes” versus “no”/“unsure,” with “no” and “unsure” combined for statistical purposes. Statistical significance was considered at *P *< 0.05 throughout the analysis.

**Table 2.** Reasons for and against adopting new ranching practice (n=706) based on participant responses to the survey question, “What are you top three reasons for adopting a new ranch management practice” and “What are your top three reasons for not adopting a new ranch management practice?

Reasons for adopting a new practice	*n*	Proportion
New practice is profitable	561	0.79
Improves animal health	550	0.78
Expected benefits outweigh effort	364	0.52
Would limit government involvement	192	0.27
Improves my quality of life	158	0.22
Improves environmental health	129	0.18
Recommended by an organization	67	0.09
Recommended by neighboring rancher or friend	62	0.09
Reasons for not adopting a practice		
Costs outweigh the benefits	*n*	Proportion
Practice does not fit into current production system	520	0.74
Practice does not align with values	418	0.59
Requires audits	311	0.44
Takes too much time	269	0.38
Benefits last lest than 5 yr	217	0.31
Requires certification	108	0.15
Requires re-certification	68	0.10
Other reason	50	0.07

To identify rancher’s perceptions of sustainability, a word cloud analysis (Cidell, 2010) was used on the open-ended question, “What does sustainability mean to you?” Word clouds provide visual representations of word frequency in a passage of text. As in Roche et al. (2015), individual responses in the text were coded using an iterative coding process of summarizing and organizing text passages (Neuman, 2007; Knapp and Fernandez-Gimenez, 2009). Individually coded responses were then computed under each theme and the number of survey participants addressing each theme.

## RESULTS AND DISCUSSION

### Rancher Education: Sources and Trust in Information

**Figure 2.**
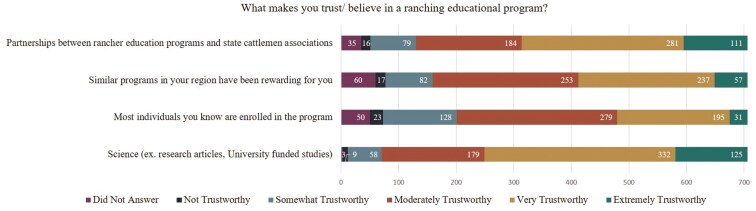
Survey responses to the question “What makes you trust/ believe in a ranching educational program?” (n = 706).

For a ranching program to be successful there needs to be a level of trust for the program to be adopted (Brain et al., 2014; Lien et al., 2017). In the current study, when survey participants were asked what made them trust or believe in ranching educational programs, 65% of participants stated they trusted (rated either very trustworthy or extremely trustworthy) science and 55% indicated they trusted rancher educational programs that partnered with state cattlemen associations (Figure 2). Forty-one percent of participants stated they would trust programs similar to those already implemented in their area that had proven beneficial for them. Lastly, only 32% of ranchers stated they would trust an educational program if most of the ranchers they knew were already enrolled in the program.

**Figure 3.**
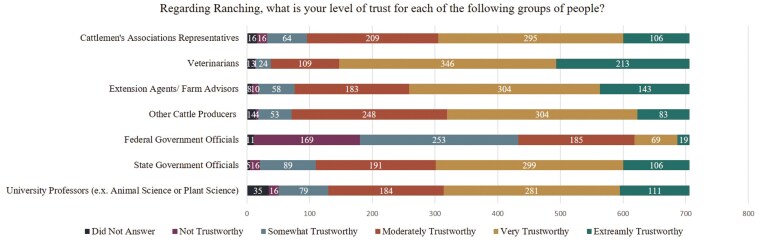
Survey responses to the question “Regarding Ranching, what is your level of trust for each of the following groups of people?” (n = 706).

When survey participants were asked who they trusted regarding information as it related to ranching, veterinarians were the most trusted group (either very trustworthy or extremely trustworthy, 79%), followed by extension/farm advisors, and other ranchers (both 65%; Figure 3). Cattlemen’s groups were trusted at a slightly lower rate of 56%. This result contrasted with Roche et al.'s (2015) work in California, where cattlemen’s groups were found to be one of the most trusted groups of individuals, and Kachergis et al. (2013) who found other ranchers to be one of the most utilized resources. The discrepancies between these results may be due to demographics; for instance, the Roche et al. (2015) study consisted of only California ranchers, compared to the current study which included ranchers from around the nation. Interestingly, despite the high level of trust in science (79%) among survey participants (Figure 2), only 55% of producers viewed university professors as a trusted group. This indicates a potential disconnect between the publication of science and the researchers who produce the science. Therefore, to disseminate science on sustainability and ranching educational programs, a University Professor on his/her own may not be as successful as veterinarians, extension/ farm advisors, or other ranchers. However, leveraging partnerships between veterinarians, extension specialists, and ranchers with University professors may improve rancher’s trust of the University academic community and increase the reach of pertinent research.

Similar to the results in Roche et al. (2015), federal government officials were the bête noire amongst the ranching community, with only 12% of ranchers trusting this group. Although this may not seem to be an obstacle for those in the corporate sector, there may be issues when trying to implement federal government funded sustainability or Scope 3 related programs. Future value chain sustainability efforts must recognize that mistrust can directly impact adoption rates of practices and programs. For example, the Natural Resource Conservation Service (NRCS) identified this lack of trust in government as a major barrier to influencing adoption of nutrient management practices among U.S. farmers and ranchers (USDA-NRCS, 2003). Despite this limitation, corporations and other stakeholders may be able to build value chain partnerships and working groups that include both ranchers and government, thereby mitigating mistrust for future program development.

**Figure 4.**
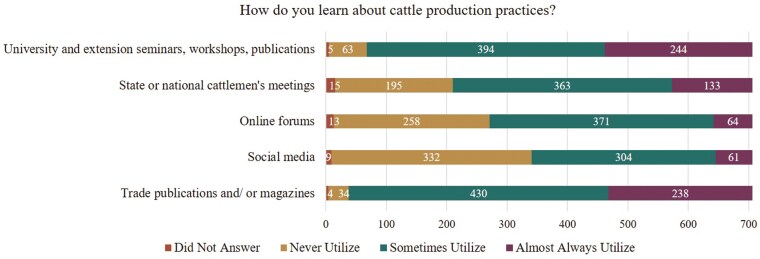
Survey responses to the question “How do you learn about cattle production practices?” (n = 706).

To effectively engage ranchers in sustainability programs, promoting sustainability related initiatives through appropriate marketing channels will be essential. Based on the present study, most producers received their ranching educational materials through printed formats and in-person delivery, such as trade publications (95%) and university/extension workshops, seminars, and publications (90%; Figure 4). On the other hand, more than 50% of ranchers also used social media and 61% use online forums. As investments in rural broadband continue to rise (USDA, 2019), and an increase in new and young ranchers (USDA-NASS, 2024), we can expect the use of these platforms will continue to grow (Muden-Dixon et al., 2018; Ghajar et al., 2019). Therefore, to effectively reach a broad range of ranchers about sustainability programs, outreach efforts should include traditional methods like mail and in-person delivery along with increased emphasis on online platforms such as social media, podcasts, and webinars.

### Reasons for and Against Adopting New Ranch Practices and Ranching Priorities

The majority (80%) of participants indicated ranching profitability was one of their primary reasons for adopting a new practice (Table 2). Although profit generally has not been considered the primary motivation for staying in ranching (Rowe et al., 2001), return on investment (ROI) has been shown to be a key factor for practice and program adoption (Didier and Brunson, 2004; Wilmer et al., 2017), as ranching operations typically function on tight margins (USDA-ERS, 2024). Therefore, sustainability programs or practices that demonstrate a reduction in time and labor while improving ROI will be more likely to be adopted and sustained (Rodriguez et al., 2009; Gedikoglu & McCann, 2012; Wade & Claassen, 2017). That said, conservation initiatives have proven to be successful even when the ROI was not direct or even short term. For example, from 2010 to 2015 the Sage Grouse Initiative collaborated with 1,500 landowners across 11 Western states to improve habitat for Sage Grouse while also improving ranching operations (USDA-NRCS, 2024). This initiative was a voluntary collaboration between ranchers and government to enhance Sage Grouse habitat to prevent the bird from being listed as an endangered species. By improving habitat and preventing the bird from being listed as endangered, ranchers were not encumbered by stringent endangered species regulation. This program was not only a successful conservation effort, but had long-term financial impacts on rancher’s license to operate^3^ . These types of grass-roots initiates continue to garner success due to communication, common goals, and ranching relationships such as the (BIOMETRICS TASK FORCE ARLINGTON VA, 2009 in northern Nebraska. Beef value chain stakeholders should evaluate success stories such as Working Lands for Wildlife and the Sandhill's Task Force on what practices and principals have true value to both the ranchers and the ranching ecosystem.

Following profitability, animal health was the second most selected reason for adopting a new practice (78% of all survey participants). Animal health has been directly tied to ranch productivity and profitability (Thornton, 2010), which in turn contributes to long-term operation viability (Schulz et al., 2017). This connection between animal health and ranch longevity may explain the success of initiatives like the Beef Quality Assurance Program (BQA; Klopatek et al., 2022), which focused on implementing practices that optimized cattle health and welfare. In light of this, sustainability programs that incorporate animal health and performance may be more likely to see broader adoption. Conversely, unlike most corporate sustainability initiatives, BQA was a voluntary program and did not directly link to incentives. The program relied on the foundation that what was better for the animal was better for the entire beef industry.

The top reason for not adopting a new practice was “costs outweigh the benefits” (74% of participants; Table 2). Changes in management practices for either soil health or carbon reductions often come with costs at the ranch-level that are frequently overlooked or misunderstood at the industry level. For example, Dyer et al. (2021) found that for the time it would take for forage benefits to be realized from conservation practice adoption (such as rotational grazing), the investment and time required were too great for many ranches to break even. In essence, the time investment and costs were insurmountable to practice adoption without additional financial assistance. This true cost of practice adoption may explain, at least in part, the lack of participation in conservation programs, even for those with perceived environmental and economic “win-wins” (Chen et al., 2023). This lack of insight on the true costs for administering potential "win-wins" on ranching operations can make implementing and incentivizing ranching sustainability programs particularly challenging. To help alleviate this confusion more research needs to specifically investigate both the environmental outcomes of practices as well as the short-term and long-term costs of adopting these new ranching practices.

Unlike with practice costs, neither initial certification nor re-certification was selected by participants as a major barrier for adopting a ranch practice or program, with less than 15% of participants selecting certification as a reason not to adopt (Table 2). Ranchers may not have perceived certification as an obstacle to adopting a practice-- for them, certification may have been viewed as an economic or educational opportunity, rather than a burden. An example of a certification program that has been viewed favorably by the cow-calf sector has been the Beef Quality Assurance certification (Klopatek et al., 2022). The major reason ranchers chose to participate in this program was because they believed BQA improved the health and welfare of their cattle (Klopatek et al., 2022). In the current survey, close to 29% of participants where in a value add certification program (Table 1), such as Verified Source and Age or Organic. Overall, the lack of ranching resistance towards certifications and high participation in certification programs provides insight for downstream industry partners who may be interested in creating value chain sustainability program or labels that require certification. Nonetheless, for a certification program to go as far as creating a value added sustainability label, it would be judicious for corporations to first determine if their customers would be willing to pay for said label.

One of the most surprising results from the survey was what ranchers identified as their top ranching priority and principal concern, with over 78% of ranchers selecting “improving or maintaining ranch publicity or public image” (n = 432). When this question was further investigated to determine if ranching demographics influenced participants primary response, no variable was identified as significant (*P* > 0.05), indicating public image was a universal concern across the ranching community. This finding aligns with previous survey results. In Roche et al. (2015) when 100 California ranchers were surveyed, 33% of participants selected “society's negative perceptions of the beef industry” as a threat to their operations. Criticism of animal agriculture has been well documented (Hannan, 2020, Morris and Jacquet, 2024) and has continued to escalate. On both a national and global scale, public rhetoric and concerns about the environmental impacts from beef production (Richter et al., 2020; Waite et al., 2022) may be impacting producers to the point where they are concerned about their business longevity. Ranching educational and sustainability programs that help ranchers tell their stories in positive and effective ways may appeal to the ranching community and increase adoption rates. In addition to maintaining ranch public image, survey participants also selected reducing additional supplementation (n = 428), improving/ maintaining herd genetics (n = 139), improving/maintaining forage production (n = 59), improving/ maintaining fertility rates (n = 50), improving/maintaining cow nutrition (i.e., body condition score (n = 45)), and improving/maintaining herd health (n = 33) as their top ranching priorities.

### Grazing Management Plan: Factors in Adoption and Plan Construction

A grazing management plan (GMP) is a strategic, adaptable tool that outlines the management and monitoring of grazing practices to achieve desired outcomes, such as optimizing livestock production as well as enhancing ecological health (Budd and Thorpe, 2009; Roche et al., 2015; Swanson et al., 2015). With the continual increase in the degradation and conversion of United States grazing lands (Reeves and Mitchell., 2012; Gage et al., 2016) implementing a GMP on ranches has become a principal interest for stakeholders in the beef value chain. Although a rancher's GMP may not guarantee outcomes or provide the same output to stakeholders as “MMRV"^4^ , they do provide the structure for ranchers to help monitor and improve grazing land ecology and productivity. As such, GMP could be integrated into stakeholder’s sustainability culture and be used as a foundational metric for creating sustainability programs. One stakeholder who has driven the messaging on GMP has been the USRSB. In 2022, the USRSB set a target for the entire beef value chain to achieve 385 million acres covered by written GMP by 2050 (USRSB, 2023). At the time of the GMP goal announcement, the USRSB did not stipulate what constituted a minimum threshold of information to be considered a GMP to meet the goal. However, the USRSB has since provided guidance and structure on what should be incorporated within a GMP including but not limited to acres grazed, species variation in grazed lands, pasture rotations, stocking densities, and rest and recovery periods (USRSB, 2023). Understanding what percentage of ranchers maintained a GMP, what was incorporated within a plan, and what motivated ranchers to produce a GMP may aid the beef value chain in increasing adoption rate and functionality of GMP. Once a baseline can be established for rate of GMP adoption, researchers can then take the needed next step to determine how various GMP have effected rancher viability and grazing ecosystems across the country.

In the present survey, 77% of participants (n = 545) reported they maintained a GMP. These results were similar to those found by Banwarth et al. (2023) who determined 81% of ranchers surveyed in California maintained a GMP. When survey participants were asked to select why they maintained a GMP from a list of multiple choice answers (5 choices total including other) the top reasons for GMP adoption included; 1) to ensure the productivity of the herd (n = 432), 2) to conserve resources (n = 339), 3) Improve family/ business partner communications (n = 105), 4) other (n = 34) and 5) mandated by lease (n = 29).To determine if some GMP were influenced by specific demographic factors (Table 3), a logistic regression was run. The analysis revealed that ranchers who were older, had a succession plan, lived in Region 6 (Southwest and California), participated in stockmanship and stewardship training, and had participated in a land assistantship program were more likely to have a GMP (*P* < 0.05; Table 3). Notably, the presence of a succession plan increased the likelihood of the survey participants maintaining a GMP. This result was consistent with Lubell et al. (2013), where ranchers with longer time horizons, i.e., those with succession plans, were more likely to see an increase in the value of sustainability initiatives over time. Therefore, ranchers with longer time horizons were expected to be associated with conservation and sustainability initiatives compared to those with shorter time horizons. In terms of age, younger producers may not know the advantages of having a GMP and thus may not have invested the resources into having one. For location, federal grazing areas (both Bureau of Land Management and the US Forest Service) maintain various guidelines for grazing sites and this may be why Region 6 (Southwest and California) with a high concentration of federal grazing areas, was more likely to have GMP compared to Region 1. Finally, many landowner assistance programs, such as the USDA Environmental Quality Incentives Program, require GMP to receive program funding; therefore, it was not surprising that ranchers in land assistantship programs would be more likely to have a GMP.

**Table 3.** Variables influencing rancher adopting of grazing managing plans (*n* = 740). Effects were analyzed using individual logistic regressions for the question “Do you have a grazing managing plan” (yes or no/unsure response categories).^1^^,^^2^

Variable	Odds Ratio	Std. Error	p-Value
Age	1.28	0.11	<0.01
Male	0.64	0.15	0.06
Region 1	0.84	0.21	0.50
Region 6	0.55	0.14	0.02
Has succession plan	1.74	0.33	<0.01
Multi-generational	1.00	0.22	0.95
Years ranching	0.94	0.90	0.58
Head of cattle	1.00	<0.001	0.44
Number of grazed acres	1.00	<0.001	0.29
Landowner assistance program	1.78	0.11	<0.01
Percent of income from cattle	1.05	0.32	0.59
Has stockmanship and stewardship training	1.61	0.48	0.02

^1^Answers included yes and no or unsure (no and unsure were combined for analysis purposes).

^2^In total ranchers with a grazing management plan totaled 545 yes, 131 no, and 28 survey takers selected unsur

Maintaining a grazing management plan in a written format can enable a  rancher to track progress, audit, and communicate the dynamic nature of grazing management over time (Jablonski et al., 2024). Furthermore, maintaining a written GMP can be valuable for monitoring practice inputs and outputs for sustainability program initiatives. The results of the present survey indicated that although 77% of ranchers maintained a GMP, only 38% (n = 207) stated their plan was written down. This result was similar to Bruno et al. (2022) where 55% or ranchers in their survey reported having an informal/unwritten ranch management plan and only 19% reported having a formal/written plan. In the current study, when a logistic regression was run to determine what factors influenced (Table 4) maintaining a written GMP, the factors were similar to those for having a GMP in any format. Specific demographic factors included ranchers who were older, had a succession plan, lived in Region 6 (Southwest and California), participated in stockmanship and stewardship training, had a land assistantship program succession plan, and maintained an increased number of grazed acres (*P* < 0.05; Table 3). The number of grazed acres may have contributed to increasing the likelihood of having a written GMP due to larger ranches having greater access to resources (Roche et al., 2015). Specifically, greater access to educational resources and a greater capacity to transform a mental GMP into a written plan. In addition, with more acres to manage there will be an inherent increase in complexity, possibly increasing the perceived benefit for maintaining a GMP in a written format. In terms of what ranchers incorporated into GMP (either written down or not), 42% stated their family members provided input (n = 230), 42% stated they utilized a technical service provider (n = 225) and 33% of ranchers stated they updated their plan every one to two years (n = 181). These results demonstrated ranchers were dynamic in their planning and overall approach to grazing management. With over a third of producers receiving input on GMP from technical service providers (TSP), for companies that want to develop sustainability initiatives with a grazing component, it may be advisable for these organizations to also consult TSP. There are currently multiple successful state and university extension programs that employ TSP across the country. Rather than reinventing the wheel, corporations and other beef stakeholders may want to collaborate with local and state extension agencies to increase the adoption of and help ensure the long-term effective utilization of a GMP and sustainability related initiatives.

**Table 4.** Variables influencing rancher adopting of a written grazing managing plans (*n* = 545). Effects were analyzed using individual logistic regressions for the question “If you have a grazing management plan, is it written down (yes or no/unsure response categories).^1^^,^^2^

Variable	Odds Ratio	Std. Error	p-Value
Age	0.78	0.07	<0.01
Male	1.36	0.30	0.15
Region 1	0.67	0.15	0.08
Region 6	0.69	0.17	0.15
Has succession plan	1.54	0.32	0.04
Multi-generational	1.00	0.11	0.02
Years ranching	1.00	0.10	0.97
Head of cattle	0.99	<0.001	0.74
Number of grazed acres	1.00	<0.001	<0.01
Landowner assistance program	1.55	0.30	0.02
Percent of income from cattle	0.90	0.10	0.31
Has stockmanship and stewardship training	1.82	0.34	<0.01

^1^Answers included yes and no or unsure (no and unsure were combined for analysis purposes).

^2^In total ranchers with a grazing management plan totaled 320 yes, 200 no, and 25 survey takers selected unsure.

### What Sustainability Means to Ranchers

Although sustainability may have different definitions and metrics across the beef value chain, there will need to be both recognition and alignment between corporations and ranchers’ pathos on sustainability. Ranchers have been the foundation of the beef value chain and their perspectives on sustainability will be pivotal for building successful beef sustainability programs. In the present study, 114 participants answered the open-ended question “What does sustainable ranching mean to you?” (Table 5; see graphical abstract). The majority of participants (65%) associated sustainable ranching with environmental health or natural resources. This result was consistent with other studies that found the majority of ranchers prioritized and viewed environmental health and natural resource conservation as “win-wins” for their ranching operations (Roche et al, 2015 and; Kachergis et al., 2013). In the present study, 49% of respondents noted economics or profitability in their definitions of sustainable ranching. Ranching has been and will continue to be an agricultural business. Without profitability, the business of ranching and ranch succession becomes increasingly difficult. Therefore, to build successful sustainability programs corporations and other beef industry stakeholders will need to invest, understand, and communicate how specific farm-level sustainability practices impact rancher profitability.

**Table 5.** Survey responses to open ended question “What Does Sustainability Mean to You?” (n = 114). Individual responses organized by subject categories.

Subject Category^1^	Count	Percent
Environmental health and natural resources	74	0.65
Economics and profitability	56	0.49
Time Horizon- continuation of ranch	49	0.43
Animal health and welfare	38	0.33
References to family	22	0.19
Less government and less regulation	10	0.09
Consumer preference	9	0.08

^1^Depending on response length and subjects listed, individual survey responses could include one or all subject categories in their definition of sustainable ranching.

 Although profitability was viewed as the number one reason to adopt a new sustainability practice, the results of the present sustainability question demonstrated how ranch profitability was not a stand alone concept. For example, 60% the producers who listed economics and/or profitability as part of ranch sustainability also included environmental health and/or natural resources (n=34) in their definition. These results largely indicated that ranchers linked profitability and environmental health as part of maintaining a sustainable ranch. When expanding on ranchers definition of sustainability and environmental health a contrarity between corporations and ranchers was revealed. Neither climate or GHG was listed by any of the 114 respondents. This result contrasts with many in the beef value chain who have publicized GHG reduction goals including beef packers, retailers, USRSB, and the National Beef Cattlemen’s Association. This discrepancy between ranchers and value chain members may be due to the fact that most corporate goals and aspirations were instated soon after this survey was distributed. Nonetheless, limited data has been available to determine if American ranchers would incorporate GHG into their sustainability definitions even with the current corporate GHG reduction goals and aspirations.

More than 40% of ranchers included a time-horizon (as defined by Lubell et al., 2013) in their definition of sustainability (n = 49), emphasizing the importance of long-term ranch viability. One of the most mentioned time horizon variables was passing along the ranch to family. In the United States, where more than 96% of farms and ranches are family-owned (USDA-NASS, 2021), family plays a pivotal role in business structure and business succession. Sustainability program initiatives who lack this time horizon component may fall flat and be difficult to accumulate interest. Future rancher related sustainability programs may find it prudent to include time horizon aspects into their programs in order to increase matriculation and retention. Furthermore, with the average age of ranchers increasing, time horizon will likely continue to play an increasingly critical role in ranch decision-making (Lubell et al., 2013).

Following time-horizon, 33% of survey participants (n = 38) included animal health and welfare as part of their definition for sustainable ranching. Although only 1/3 of the sustainability definitions included animal health and welfare, improved animal health and welfare was the second most common reason for ranchers adopting a practice (Table 2). With continued adoption of Beef Quality Assurance, and increased animal health scrutiny throughout the value chain, animal health and welfare cannot afford to be overlooked in value chain sustainability initiatives.

Overall, the results from the question on sustainability demonstrate that, to ranchers, sustainability is not myopic and true ranch sustainability incorporates a myriad of topics including natural resource preservation, economics, ranch longevity, family, and animal health.

## CONCLUSION

With the increasing pressure on food corporations to reduce Scope 3 emissions and source from sustainable value chains, the interest in building sustainability focused programs within the cow-calf sector is expected to grow. The cow-calf sector has opportunities to both reduce emissions and provide ecosystem services (FAO, 2023; Pelton et al., 2024), but ranching operations are incredibly diverse in size, management, and region. Due to the diverse nature of cattle sector, companies face an uphill battle navigating how to meet corporate sustainability goals while connecting to ranchers’ true sustainability needs. In the present study, one factor that was consistent among ranchers was their concern about public perception of the beef cattle industry. The continuing concern with beef’s environmental impact may have begun to take a toll on beef producers to the point where they are concerned about business viability. If a sustainability program could aid in educating the public to improve ranch image, this may be one way to increase rancher involvement in a sustainability program.

Due to the fragmentation of the beef industry, it may be advisable for companies to follow the lead of previously successful beef educational programs such as Working Lands for Wildlife and the Beef Quality Assurance program. These programs were voluntary based and addressed the immediate needs of the environment, animal health, and the rancher, building positive outcomes across the entire value chain. The current study presented one such cross-sectional strategy corporations and stakeholders could lean into, the incorporation of GMP into sustainability programs. While 77% of ranchers stated they maintained a GMP, only 38% of ranchers cited their GMP was written down, illustrating an opportunity to increase the total number of written GMP. Although grazing management does not guarantee ecological or economic outcomes, a GMP could be a positive step in building more resilient, thereby sustainable, beef value chains. In that aspect, corporations can be a part of the solution by funding technical service providers to increase the number and effectiveness of a written GMP. This funding can also ensure that a basic minimum threshold for what is needed to be in a GMP can be included, such as pasture rest, recovery, stocking density, and species diversity.

Finally, beef industry stakeholders would also benefit by aligning their sustainability definitions and programs with those definitions and beliefs held by the ranching community. Based on the survey results, ranchers viewed sustainability as the nexus of animal health and performance, preservation of natural resources, and long-term economic viability. Although appropriate reimbursement would be foundational for program participation, based on rancher feedback, sustainability programs that also incorporate aspects of family, time horizon, and natural resource conservation, may benefit from increased program matriculation. To continue to build on these survey results future research will be needed to investigate the short- and long-term costs of any sustainability related program and practice adoption, how GMP can and cannot improve performance and profitability, and how communication platforms can be utilized to demonstrate the impact ranching communities have on grazing lands. Through these type of research initiatives both trust and understanding can grow between beef value chain members. For at the end of the day, for true partnerships to be successful trust, understanding, and communication will be essential.

## Supplementary Material

txaf045_suppl_Supplementary_Materials

## Footnotes

1 C-Suite includes the senior executives and managers whose job titles start with “C,” such as the chief executive officer (CEO) and chief financial officer (CFO)

2 For the purposes of this research article the term ranchers and producers are interchangable. Ranchers/producers were considered to be individuals who owned and/ or managed cattle. 

3 The “license to operate” is a business's permission to operate within a given area, subject to regulation. License to operate can also be a measure of how well a company is accepted by the public and other stakeholders.

4 Measurement, Monitoring, Reporting, and Verification is a multi-step process used to account for greenhouse gas (GHG) emissions across value chains. Companies offer MMRV services to other companies with a vested interested in tracking their GHG inventories.  
