# Correction to: Filling the sustainability gap: what beef industry stakeholders can learn from ranchers on new practice adoption, grazing management plans, and sustainability

**DOI:** 10.1093/tas/txag003

**Published:** 2026-02-24

This is a Correction to: S C Klopatek, A M Cantwell, L Roche, J W Oltjen, Filling the sustainability gap: what beef industry stakeholders can learn from ranchers on new practice adoption, grazing management plans, and sustainability, *Translational Animal Science*, Volume 9, 2025, txaf045, https://doi.org/10.1093/tas/txaf045

In the originally published paper, the formatting had been errored during production steps in Table 2 and this interrupted data presentation from midway onwards.

Table 2 should read:

**Table 2.** Reasons for and against adapting new ranching practice (n=706) based on participant responses to the survey question, “What are you top three reasons for adopting a new ranch management practice” and “What are your top three reasons for not adopting a new ranch management practice?

Reasons for adopting a new practice	*n*	Proportion
New practice is profitable	561	0.79
Improves animal health	550	0.78
Expected benefits outweigh effort	364	0.52
Would limit government involvement	192	0.27
Improves my quality of life	158	0.22
Improves environmental health	129	0.18
Recommended by an organization	67	0.09
Recommended by neighboring rancher or friend	62	0.09

Reasons for not adopting a practice	*n*	Proportion
Costs outweigh the benefits	520	0.74
Practice does not fit into current production system	418	0.59
Practice does not align with values	311	0.44
Requires audits	269	0.38
Takes too much time	217	0.31
Benefits last lest than *5* yr	108	0.15
Requires certification	68	0.10
Requires re-certification	50	0.07
Other reason	19	0.03

instead of:

**Table 2.** Reasons for and against adopting new ranching practice (n=706) based on participant responses to the survey question, “What are you top three reasons for adopting a new ranch management practice” and “What are your top three reasons for not adopting a new ranch management practice?

Reasons for adopting a new practice	*n*	Proportion
New practice is profitable	561	0.79
Improves animal health	550	0.78
Expected benefits outweigh effort	364	0.52
Would limit government involvement	192	0.27
Improves my quality of life	158	0.22
Improves environmental health	129	0.18
Recommended by an organization	67	0.09
Recommended by neighboring rancher or friend	62	0.09
Reasons for not adopting a practice		
Costs outweigh the benefits	*n*	Proportion
Practice does not fit into current production system	520	0.74
Practice does not align with values	418	0.59
Requires audits	311	0.44
Takes too much time	269	0.38
Benefits last lest than *5* yr	217	0.31
Requires certification	108	0.15
Requires re-certification	68	0.10
Other reason	50	0.07

Correction has been made in the article.

