# Supplementary material for: Filling the sustainability gap: what beef industry stakeholders can learn from ranchers on new practice adoption, grazing management plans, and sustainability
Source: Transl Anim Sci. 2025 Apr 16;9:txaf045. doi: 10.1093/tas/txaf045 (PMC12199235; doi:10.1093/tas/txaf045)
Supplement: txaf045_suppl_Supplementary_Materials [file txaf045_suppl_supplementary_materials.docx]

Ranch Management Practices

Start of Block: Section 1: Rancher Demographics

Q59

Q36 Ranchers are the foundation of the beef supply chain.  Therefore, for a rancher educational program to be effective it needs to be designed for RANCHERS by RANCHERS.  That’s why we are asking for your help. We want to learn from you, the rancher, on what matters to you when ranching.  All your answers will be *completely anonymous* and the information collected will be reported as summaries and not connected to your name or address in anyway. Results from the survey will be complied and analyzed at the University of California Davis, in the Animal Science Department. Data collected will be used to improve current rancher educational programs.

Q1
To start the survey, we would like to know about your unique ranching experiences and how they have influenced the way you manage your ranch and ranching goals. Please answer the following ranching operation demographic questions.

What is your current age?

- 18-30 (1)
- 30-40 (2)
- 40-50 (3)
- 50-60 (4)
- 60-70 (5)
- 70 and older (6)

Q2 How many years have you personally been ranching? (Since you were 18 years old)

- 0-5 Years (1)
- 5-10 years (2)
- 10-20 years (3)
- 20-30 years (4)
- More than 30 years (5)

Q57 What is your primary zip code?

________________________________________________________________

Q3 What is your gender?

- Male (1)
- Female (2)

Q4 On average (over the last 5 to 10 years), how many animals of each livestock type graze on land you own or personally lease?

|  | Total Number of Animals (1) |
| --- | --- |
| Number of cows and yearling heifers (1) |  |
| Number of stockers (2) |  |
| Number of bulls (3) |  |
| Number of sheep (4) |  |

Q5 On average (over the last 5 to 10 years), what are the reproductive outcomes for your cattle?

|  | Number (1) |
| --- | --- |
| Number of cattle exposed to a bull / artificially inseminated (AI) (1) |  |
| Number of calves born (2) |  |
| Number of calves weaned (3) |  |
| Length of the breeding season (days) (4) |  |
| Average weaning age (days) (5) |  |
| Average weaning weight (pounds) (6) |  |

Q6 Please describe your grazing land.

|  | Acres of Land (1) |
| --- | --- |
| Total acres personally owned for grazing (1) |  |
| Total privately owned acres leased for grazing (2) |  |
| Total public acres leased for grazing, public (ex. Forest Service land) (3) |  |
| Total irrigated acres for grazing (owned or leased) (4) |  |
| Total acres managed for grazing (owned, leased, and irrigated) (5) |  |

Q7 Are you a first or multi-generation rancher?

- First generation rancher (1)
- Multi-generation rancher (2)

Q8 Do you have a succession plan for your ranch that identifies a strategy for keeping land in ranching in the future?  (ex. pass on to family for ranching)

- Yes (1)
- No (2)
- In progress (3)
- Not applicable (Do not own land) (4)

Q10 What is your percent of your total income coming from the ranch (beef cattle only)?

- 1-25% (1)
- 26-50% (2)
- 51-75% (3)
- 76%-100% (4)

Q12 Does your operation include other activities that affect land management? (Check all that apply)

- Other agricultural production (1)
- Passive recreation (ex. hiking, birding, horseback riding) (2)
- Conventional energy development (ex. oil, coal, natural gas) (3)
- Extractive Recreation (ex. hunting, fishing) (4)
- Alternative energy development (ex. solar, wind, biofuel) (5)
- Special Events (ex. weddings, parties) (6)
- Other (7)

End of Block: Section 1: Rancher Demographics

Start of Block: Section 2: Program Participation

Q53 **Please answer the following questions regarding your participation in ranching programs and current program involvement.**

Q13 Do you participate in any ranching certification programs, if so which ones? (Check all that apply)

|  | Program (1) |
| --- | --- |
| I do not participate in a ranching certificate program (1) |  |
| Humanely Raised (2) |  |
| 100% Grass-Fed or Grass-Finished (3) |  |
| All Natural (4) |  |
| Beef Quality Assurance (BQA) (5) |  |
| Certified Organic (6) |  |
| Verified Source and Age (7) |  |
| Non-hormone Treated Cattle (NHTC) (8) |  |
| Global Animal Partnership (GAP) (9) |  |
| Other (10) |  |

Q14 Do you participate in any government landowner assistance program? [ex. USDA or Natural Resource Conservation Service (NRCS), such as the Environmental Quality Incentives Program (EQIP), Conservation Stewardship Program (CSP)]

- Yes (If yes, please fill in program below) (1) ________________________________________________
- No (2)

Q15 Which of the following best describes your participation in the Beef Quality Assurance (BQA) program?

- I have not participated in BQA (5)
- I went to a BQA program, but I never became BQA certified (4)
- I was BQA certified at one time, but never re-certified (3)
- I am currently certified (I first enrolled in BQA within the last 3 years) (1)
- I am currently certified (I have been in BQA for over 3 years and I have re-certified at least once) (2)

Skip To: Q21 If Which of the following best describes your participation in the Beef Quality Assurance (BQA) prog... = I have not participated in BQA

| 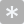 |
| --- |

Q17 If you participated in Beef Quality Assurance (BQA), what were your top 3 reasons for joining the program? (Please select up to **3 choices** from the following options.)

- BQA animals fetch a higher price (1)
- Neighbors/competitors were also performing BQA practices (2)
- Improve animals’ health and welfare (3)
- Increases longevity of your operation (4)
- Believed voluntary participation would prevent regulatory requirements (5)
- Reputation of my operation is greater when animals are a part of BQA (6)
- Consumer perceptions/demand concerns about animal welfare (7)

Q58 If you have participated in BQA, do you believe that adopting BQA practices was beneficial to your ranching operation?

- Extremely beneficial (1)
- Very beneficial (2)
- Beneficial (3)
- Somewhat beneficial (4)
- Not beneficial (5)

Q20  If you have participated in BQA, are there any sections in the BQA guidelines that you did not find feasible or helpful for your operation? Please write your answer below and explain why the section(s) were not feasible and/or helpful. (Sections include, antibiotic/vaccination procedures, weaning practices, and transportation procedures).  Please write your answer below.

________________________________________________________________

Q21 If you have **NOT**participated in Beef Quality Assurance (BQA),  please select from the following statements below as to why you did not join BQA. (Check all that apply)

- I was not aware of the BQA program (1)
- No BQA certification opportunities in my area (2)
- Time commitment too high (3)
- Not enough financial reward (4)
- BQA practices do not make sense or are confusing (5)
- My operation exceeds BQA standards and so I do not believe it is necessary (6)
- Practices do not fit my operation goals or management strategies (7)
- Other (If other, please explain) (8) ________________________________________________

End of Block: Section 2: Program Participation

Start of Block: anching Education Programs In order to improve current Rancher Educational progr

Q39 **In order to improve current rancher educational programs, we need to know what educational resources are most useful and what information is most valuable to you within these programs. Please fill out the following questions regarding rancher educational programs.**

Q23 Please choose from the list below on what makes you trust/believe in an educational program? Please check the boxes below.

|  | Not Trustworthy (1) | Somewhat Trustworthy (2) | Moderately Trustworthy (3) | Very Trustworthy (4) | Extremely Trustworthy (5) |
| --- | --- | --- | --- | --- | --- |
| Science (ex. research articles, University funded studies) (1) |  |  |  |  |  |
| Most individuals you know are currently enrolled in the program (3) |  |  |  |  |  |
| Similar programs in your region have been rewarding for you (5) |  |  |  |  |  |
| Partnerships between rancher education programs and state cattlemen associations (4) |  |  |  |  |  |

Q24  When discussing/learning about ranching, what is your level of trust for each of the following groups of people. Please check the boxes below.

|  | Not Trustworthy (1) | Somewhat Trustworthy (2) | Moderately Trustworthy (3) | Very Trustworthy (4) | Extremely Trustworthy (5) |
| --- | --- | --- | --- | --- | --- |
| University Professors (e.x. Animal Science, Range Science, Plant Science) (1) |  |  |  |  |  |
| State Government Officials (2) |  |  |  |  |  |
| Federal Government Officials (3) |  |  |  |  |  |
| Other Cattle Producers (4) |  |  |  |  |  |
| Extension Agents/Farm Advisers (5) |  |  |  |  |  |
| Veterinarians (6) |  |  |  |  |  |
| Cattlemen’s Associations Representatives (7) |  |  |  |  |  |

| 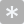 |
| --- |

Q25 Please select your 3 top reasons why you would adopt a new ranching practice. (Check up to 3 boxes that apply)

- It is profitable (1)
- It improves animal health (3)
- Would limit government involvement (5)
- Expected benefits greatly out weigh cost and effort to implement (2)
- It improves environmental health (4)
- It improves my quality of life (6)
- Another rancher/ranching friend has recommended the program (7)
- It is recommended by national/state/local cattlemen’s organization(s) (8)

| 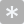 |
| --- |

Q27 Please select your **3 top reasons** why you would **NOT** adopt a new ranching practice. (Check up to 3 boxes that apply)

- It would require more time investment (1)
- Does not fit your production system (2)
- Requires certification (3)
- Requires re-certification (4)
- Requires audits (5)
- Expected cost outweighs expected benefits (6)
- Benefits are short term (5 years or less) (7)
- Does not align with your values (8)
- Other (If other, please explain) (9) ________________________________________________

Q26  How do you learn about cattle production practices? Please check the boxes below.

|  | Never Utilize (1) | Sometimes Utilize (2) | Almost Always Utilize (3) |
| --- | --- | --- | --- |
| Trade publications and/or magazines (ex. cattlemen’s association magazines, Drovers Daily, Farm and Ranch, etc.) (1) |  |  |  |
| Social media (ex. Facebook, Twitter, Instagram, etc.) (2) |  |  |  |
| Online forums (ex. web seminars/ training sessions) (3) |  |  |  |
| State or National cattlemen’s meetings (4) |  |  |  |
| University and extension seminars, workshops, publications (5) |  |  |  |

Q28 Where do you give vaccine/ hormone/ antibiotic injections for beef cattle? You may choose more than one location for each injection type.

|  | Shoulder (1) | Rump (2) | Tailhead (3) | Neck (4) | Other (5) |
| --- | --- | --- | --- | --- | --- |
| Antibiotics (1) |  |  |  |  |  |
| Vaccines (2) |  |  |  |  |  |
| Reproductive hormones (ex. estroplan, P.G. 600) (3) |  |  |  |  |  |

Q29 When managing your herd, what procedures do you use/perform? (Check all that apply)

- Fence line wean, utilizing a fence to separate the cow from the calf (1)
- Waiting 45 days after weaning to ship calves (2)
- Ship calves immediately or very shortly after weaning (3)
- Ship calves 7 to 45 days after weaning (4)
- Castrate before 3 months of age (5)
- Castrate after 3 months of age (6)
- Darting, using remote devices to give injections (7)
- Established a herd health development program with your vet (8)
- Other methods (If other, please explain) (9) ________________________________________________

Q32 Have you ever participated in stockmanship/stewardship training?

- Yes (1)
- No (2)
- Unknown (3)

Q40 When managing cattle,  how important is it for you to use low stress livestock handling techniques?

- Extremely important (1)
- Very important (3)
- Important (4)
- Somewhat important (5)
- Not important (6)

End of Block: anching Education Programs In order to improve current Rancher Educational progr

Start of Block: Grazing and Cow-calf Management

Q34 **In order to help create educational programs that help you reach your cow-calf/stocker operation goals, we would like to know what you think is most important when it comes to managing your herd. Please answer the questions below in regard to your heard genetics and grazing management.**

Q35 When you buy breeding stock, have ranching educational programs influenced your purchasing decisions?

- Yes (1)
- Maybe/Unsure (2)
- No (3)

Q43 Do you have a grazing management plan?

- Yes (1)
- No (2)
- Unsure (3)

Skip To: Q46 If Do you have a grazing management plan? = No

Skip To: Q46 If Do you have a grazing management plan? = Unsure

Q51 If you have a grazing management plan, what features are included? (Check all that apply)

- Written down (1)
- Updated every 1 to 2 years (2)
- Family provides input (3)
- Technical services provide input, such as farm advisers, extension specialists, or NRCS specialists (4)
- Other (5) ________________________________________________

Q45 If you have a grazing management plan, what motivated you to develop a grazing management plan? (Check all that apply)

- Mandated via a lease (1)
- To ensure productivity of herd (2)
- Resource conservation (3)
- Improve family/business partner communications (4)
- Other (If other, please specify) (5) ________________________________________________

Q46 From the list below, please rank from 1-7 your top goals when managing your cow-calf herd. With 1 being the highest priority and 7 being your lowest priority.

______ Improving/maintaining cow nutrition (ex. body condition score) (2)

______ Improving/maintaining fertility rates (4)

______ Improving/maintaining forage productivity (5)

______ Improving/maintaining herd health (6)

______ Improving/maintaining genetics of the herd (7)

______ Improving/maintaining ranch publicity/ public image (8)

______ Reducing additional supplementation (ex. "Haying out") (9)

Q47 As part of a voluntary rancher education program you might join, would you be willing to (Please select all that apply):

- Individually identify each calf (1)
- Record birthdates (2)
- Record location of each animal (3)
- Record stocking densities (4)
- Record biosecurity plans (5)
- Maintain animal health records (6)

Q48 How important is it to you to manage standing forage or the amount of grass in your pasture/rangeland? (Please select **one** of the following choices below.)

- Extremely important (1)
- Very important (2)
- Moderately important (3)
- Slightly important (4)
- Not at all important (5)

Q49 Would participating in a non-mandatory "beef sustainability" rancher education program be beneficial for your operation?

- Yes (1)
- No (2)
- Unsure (3)

Q50 It is vital to hear ranchers voices on sustainability. Therefore, for the last question, please describe what "sustainable ranching" means to you.

________________________________________________________________

________________________________________________________________

________________________________________________________________

________________________________________________________________

________________________________________________________________

Q61 OPTIONAL QUESTION FOR PRIZES:

End of Block: Grazing and Cow-calf Management
